# Cenobamate use in super‐refractory status epilepticus: A report of three cases

**DOI:** 10.1002/epd2.70270

**Published:** 2026-07-04

**Authors:** Marina Romozzi, Juan Luis Alcala‐Zermeno, Antonio Funcis, Federico Tosto, Marta Piccioli, Luigi Francesco Iannone, Riccardo Maviglia, Masahito Katsuki, Yasuhiko Matsumori, Paolo Calabresi, Raffaele Iorio, Catello Vollono

**Affiliations:** ^1^ Dipartimento di Neuroscienze Università Cattolica del Sacro Cuore, Fondazione Policlinico Universitario Agostino Gemelli IRCCS Rome Italy; ^2^ Neurologia Unità Operativa Complesse, Dipartimento di Neuroscienze, Organi di Senso e Torace Fondazione Policlinico Universitario Agostino Gemelli IRCCS Rome Italy; ^3^ Comprehensive Epilepsy Center, Department of Neurology Columbia University Irving Medical Center, New York Presbyterian Hospital New York New York USA; ^4^ Dipartimento di Neuroscienze Presidio Ospedaliero Giovanni Paolo II Lamezia Terme Italy; ^5^ Neurologia Unità Operativa Complesse Presidio Ospedaliero San Filippo Neri Hospital, ASL Roma 1 Rome Italy; ^6^ Department of Biomedical, Metabolic and Neural Sciences, University of Modena and Reggio Emilia, Modena, Italy University of Modena and Reggio Emilia Modena Italy; ^7^ Dipartimento di Scienze Dell'Emergenza, Anestesiologiche e Della Rianimazione Fondazione Policlinico Universitario Agostino Gemelli IRCCS Rome Italy; ^8^ Headache Center, Department of Neurology Tominaga Hospital Osaka Japan; ^9^ Insight Science Foundation Ireland Research Centre for Data Analytics, School of Human and Health Performance Dublin City University Dublin Ireland; ^10^ Sendai Headache and Neurology Clinic Sendai Japan

**Keywords:** cenobamate, FIRES, NORSE, seizures, SRSE

## Abstract

**Objectives:**

Super‐refractory status epilepticus (SRSE) is a neurological emergency with high morbidity and mortality. Cenobamate, a novel antiseizure medication, may be helpful in managing SRSE, but evidence is limited.

**Methods:**

This retrospective case series reports the use of cenobamate as add‐on therapy in the management of three cases of SRSE.

**Results:**

Median age at status epilepticus (SE) onset was 28 years (range 27–75). The etiologies of SRSE included one case of febrile infection‐related epilepsy syndrome (FIRES), one patient with a probable genetic etiology (SCN7A variant of uncertain significance), and one case of unknown etiology. Cenobamate was introduced at a median of 27 days (range 13–103) after SE onset. Resolution of SRSE was observed after a median of 7 days (range 3–30) once cenobamate was started. Median dose of cenobamate at SRSE cessation was 25 mg/day (range 12.5–50 mg/day), and the median maintenance dose at the time of discharge was 200 mg/day (range 100–400 mg/day). Two patients died and one patient achieved functional independence. No severe medication side effects, specifically drug reaction with eosinophilia and systemic symptoms (DRESS), were observed.

**Discussion:**

Cenobamate may have a role in the management of SRSE. Further studies are needed to define the optimal timing of initiation, titration strategy, and target dose of cenobamate in the context of SE.

**Key points**

- Cenobamate may represent a promising adjunctive therapy in super‐refractory status epilepticus (SRSE).
- We report three cases of SRSE (FIRES, probable genetic etiology, and unknown etiology) treated with cenobamate, in which electroclinical improvement and cessation of status epilepticus occurred after drug introduction.
- Clinical outcomes were heterogeneous, ranging from meaningful recovery to severe disability and death, despite control of status epilepticus.
- Including the present series, a total of six cases of SRSE treated with cenobamate have been reported in the literature.
- Across all six cases, cenobamate introduction was temporally associated with the resolution of SRSE, supporting a potential therapeutic signal that warrants further systematic investigation.
- Further studies are needed to define the optimal timing of initiation, titration strategy, and target dose of cenobamate in the context of status epilepticus.

## 1 INTRODUCTION

Cenobamate has advanced the treatment of drug‐resistant focal epilepsy, demonstrating higher rates of seizure reduction and seizure freedom in clinical trials and real‐world studies.^1^, ^2^ Its use in the acute inpatient setting is limited by its slow titration, which is required to minimize the risk of drug reaction with eosinophilia and systemic symptoms (DRESS), a serious and potentially fatal allergic reaction.^1^, ^2^

Status epilepticus (SE) represents the failure of the mechanisms responsible for seizure termination, leading to an abnormally prolonged seizure.^3^ When ictal activity persists or recurs for more than 24 h after initiation of anesthetic therapy, SE is labeled super‐refractory status epilepticus (SRSE), which is associated with high mortality rates ranging between 22% and 32%.^4^, ^5^ Attempts at introducing oral antiseizure medications as add‐on treatment for SRSE management, such as perampanel^6^ and topiramate,^7^ have produced mixed results.

## 2 METHODS

To assess the evidence of cenobamate use in SRSE, we performed a comprehensive literature search in the PubMed and Web of Science databases using the terms: [(Cenobamate) AND (Super‐refractory status epilepticus) OR (new onset refractory status epilepticus) OR (Status epilepticus)] performed until March 2025 and found three cases documenting its use and safety in SRSE management.^8^, ^9^ To build on this, we report our experience using cenobamate in three consecutive SRSE cases managed at a single referral center. In our case series, cenobamate was administered via nasogastric tube or percutaneous endoscopic gastrostomy (PEG), which has similar bioavailability to intact tablet ingestion.^10^ Data were obtained from the medical record, and patients or family members were contacted by telephone to determine post‐hospitalization outcomes. All patients (or family members) provided written informed consent. Baseline characteristics and diagnostic test results are summarized in Table 1. Relevant management timelines and imaging findings are shown in Figure 1. Details on testing and immunotherapy use at our center are described in detail in the Data S1.

**TABLE 1:** Clinical characteristics and therapeutic management of three patients with status epilepticus treated with cenobamate.

	Case 1	Case 2	Case 3
Age/Sex	28‐year‐old male	75‐year‐old female	27‐year‐old female
Etiology	Unknown (possible genetic etiology due to variant of uncertain significance in the SCN7A gene)	Unknown	FIRES‐Related to Coxiella burnetii infection
Initial SE semiology	Convulsive	Non‐convulsive	Convulsive
Ictal EEG changes	Right‐hemispheric rhythmic discharges with a frequency of 2–3 Hz (spikes to sharply contoured waveforms) superimposed on fast activity and moderate to severe diffuse background slowing	Generalized spike‐and‐wave discharges with a frequency of 2.5–3 Hz	Diffuse epileptiform discharges, prevalent in right frontal‐temporal region
Brain MRI	Bilateral mesial temporal, insula, claustrum, and pulvinar T2‐FLAIR hyperintensities	Initial: Unremarkable Repeat (day 19): Bihemispheric T2‐FLAIR hyperintensities accentuated in the right frontal lobe as well as right frontal cortico‐pial changes	Initial: Unremarkable Repeat (day 10): Bilateral mesial temporal T2‐FLAIR hyperintensities
CSF features**	No WBCs, normal protein, normal glucose	Lymphocytic pleocytosis, elevated protein, normal glucose	No WBCs, normal protein, normal glucose
ASMs prior to CBM*	First: LEV (3000 mg /day), VPA (2400 mg/day)|second: PB (200 → 400 mg/day), LCM (200 → 400 mg/day)|third: PER (4 → 6 mg/day)	First: LEV (3000 mg/day) | second: VPA (1200 mg/day) | third: PB (100 → 200 mg/day)	First: VPA (1600 → 2000 mg/day), PB (50 → 300 mg/day)| second: PHT (100 → 300 mg/day)|third: BRV (100 → 300 mg/day), LCM (200 → 400 mg/day)|fourth: PER (4 → 6 mg/day)
Anesthetic infusions prior to CBM	Propofol IV (1 g 22 ml/h → 1 g 32 ml/h), midazolam (0.16 mg/kg/h)	Propofol IV (1 g 18 ml/h → 1 g 30 ml/24 h)	Propofol IV (1 g 18 ml/h → 1 g 30 ml/24 h)
Immunotherapy**	IVMP, IVIG, PLEX, ocilizumab	IVMP, IVIG	IVMP, IVIG, PLEX
Other treatments**	Broad‐spectrum antimicrobials with CNS coverage. Ketogenic diet	Broad‐spectrum antimicrobials with CNS coverage	Sulfamethoxazole‐trimethoprim, hydroxychloroquine, doxycycline
CBM start	103 days from SRSE onset	13 days from SRSE onset	27 days from SRSE onset
CBM dose	12.5 mg/day × 14; 25 mg/day × 14; 50 mg/day × 14; 100 mg/day × 14; 150 mg/day × 14; 200 mg/day × 14; then titrated to 400 mg/day ongoing	12.5 mg/day × 14; 25 mg/day × 14; 50 mg/day × 14; 100 mg/day ongoing	12.5 mg/day × 7; 25 mg/day ×7; 50 mg/day x 14; 100 mg/day ×14; 150 mg/day × 28; 200 mg/day ongoing
Propofol weaning	132 days from SRSE onset	17 days from SRSE onset	35 days from SRSE onset
ASMs at discharge from the hospital	CBM (400 mg/day), LCM (400 mg/day), LEV (3000 mg/day), PB (200 mg/day)	CBM (100 mg/day), LEV (3000 mg/day), PB (125 mg/day), VPA (1200 mg/day)	CBM (200 mg/day), LCM (400 mg/day), PB (100 mg/day), VPA (1500 mg/day)
GCS at discharge from the hospital	7	5	15

Abbreviations: ASMs, antiseizure medications; BRV, brivaracetam; CBM, cenobamate, CNS, central nervous system; FIRES, febrile infection‐related epilepsy syndrome; GCS, Glasgow coma scale; IV, intravenous; IVIG, intravenous immunoglobulin; IVMP, intravenous methylprednisolone; LCM, lacosamide; LEV, levetiracetam; PB, phenobarbital; PER, perampanel; PHT, phenytoin; PLEX, plasma exchange; SCN7A, alpha subunit of the voltage‐gated sodium channel type 7; SE, status epilepticus; SRSE, super refractory status epilepticus; T2‐FLAIR, T2‐weighted fluid‐attenuated Inversion recovery; VPA, valproic acid; WBCs, white blood cells.

**FIGURE 1:**
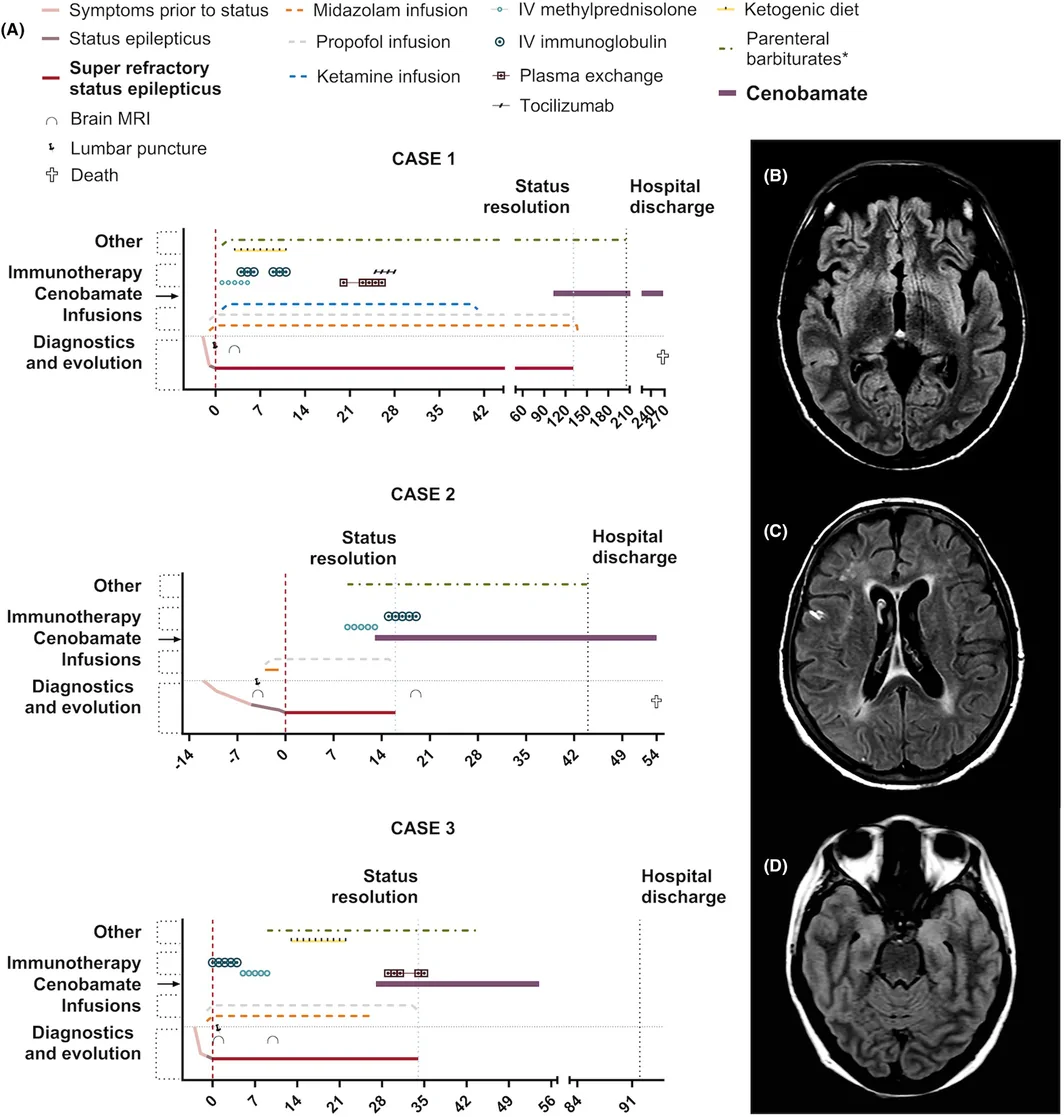
Clinical trajectories (panel A) and MRI findings (panels B–D) of patients with super‐refractory status epilepticus (SRSE) treated with cenobamate. The vertical red dotted line in the clinical trajectories represents time 0 when patients were diagnosed with SRSE. Panel B shows T2‐FLAIR hyperintense signal at the level of the left claustrum and insula as well as bilateral pulvinar hyperintensities seen in case 1. Panel C shows T2‐FLAIR hyperintensity of the periventricular bihemispheric white matter with multiple subcortical hyperintense areas and a focal area of increased signal intensity at the cortico‐pial level and underlying white matter in the right frontal lobe, seen in case 2. Panel D shows T2‐FLAIR hyperintensity of the mesial temporal lobes in case number 3. *Either intravenous or intramuscular. Upon discharge, barbiturates were transitioned to the enteral route.

### 2.1 Standard protocol approvals, registrations, and patient consents

Written informed consent was obtained from all participants (or guardians of participants) included in the case series. The study was performed in agreement with the Declaration of Helsinki. IRB approval: not applicable (retrospective case series with ≤3 patients).

### 2.2 Case 1

A previously healthy 28‐year‐old male presented with generalized convulsive SE and was initially managed with intravenous (IV) diazepam, levetiracetam, valproic acid, lacosamide, and perampanel oral suspension, under continuous EEG monitoring. Electroclinical SE persisted, requiring intensive care unit (ICU) admission and anesthetic therapy with midazolam and propofol. After 24 h, an anesthetic wean was attempted. SE recurred, prompting initiation of IV ketamine followed by parenteral barbiturates. CSF analysis was negative for pleocytosis, oligoclonal bands (OCB), viral, bacterial, and autoimmune markers (see Data S1). Brain MRI revealed bilateral mesial temporal, insular and claustrum hyperintensities (Figure 1, Panel B). Immunotherapy was administered in the following sequence: IV methylprednisolone, intravenous immunoglobulin (IVIG) 2 cycles, plasma exchange, and tocilizumab. Continuous EEG revealed 2–3 Hz right‐hemispheric rhythmic discharges with fluctuating morphology, ranging from spikes to sharply contoured waveforms, superimposed on fast activity, and moderate to severe diffuse background slowing (Figure 2, panel A). Ketogenic diet was tried for 9 days to no avail. Whole‐exome sequencing revealed a variant of uncertain significance in the SCN7A gene. Several anesthetic weans were attempted, but the patient had a recurrence of facial clonic movements. After 3.5 months from SRSE onset, cenobamate was initiated at 12.5 mg/day. Thirty days after the initiation of cenobamate (the patient had just reached the 50 mg/day dose), the patient was weaned off propofol successfully, and 1 week after, he was weaned off midazolam. The EEG showed a sustained pattern resolution (Figure 2, panel B).

**FIGURE 2:**
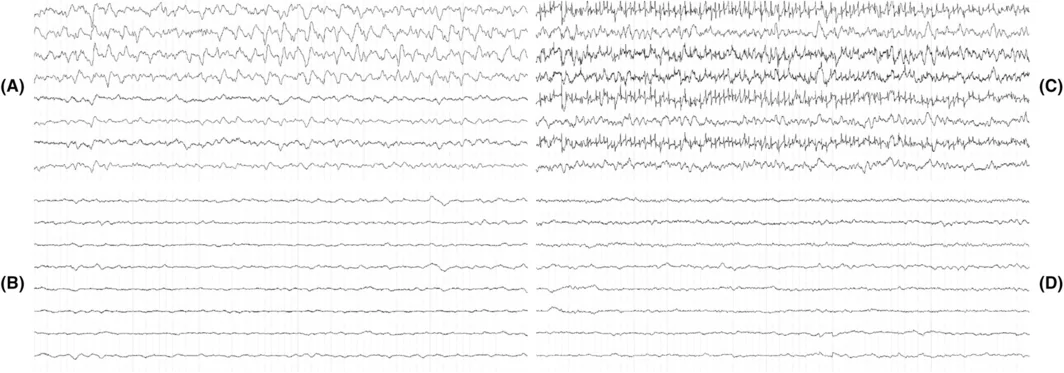
Panel A shows a continuous vEEG tracing of 2–3 Hz continuous right hemispheric rhythmic discharges with fluctuating morphology between sharp waves and sharply contoured waveforms with superimposed fast activity as well as moderate to severe diffuse background slowing. Panel B shows sustained pattern resolution after administration of cenobamate and discontinuation of IV sedatives. Panel C shows a continuous vEEG tracing of 1.5–2.5 Hz frontotemporal predominant generalized periodic discharges with morphology that fluctuates between spikes and sharply contoured waveforms with superimposed spiking (GPDs + S) occupying >20% of the recording. Given the improvement observed af ter administration of cenobamate (Panel D), this was compatible with possible electroclinical status epilepticus.^11^

Cenobamate was titrated to a 400 mg/day dose, which the patient tolerated in addition to the other antiseizure medications (levetiracetam, lacosamide, and phenobarbital). The patient was discharged to rehabilitation ‐7 months after the onset of status. At the time, he could open his eyes and had fluctuating awareness but was not able to communicate. Two months after his discharge, he passed away in the rehabilitation facility due to pneumonia.

### 2.3 Case 2

A 75‐year‐old female presented with a 3‐day history of altered mental status, fever, diffuse arthralgias, and myalgia, initially managed with azithromycin and oral corticosteroids in the outpatient setting. Her level of alertness continued to decline. The patient was evaluated in the emergency room, where she was intubated and transferred to the ICU. Continuous EEG monitoring revealed a pattern compatible with nonconvulsive SE, prompting sedation with propofol and rapid sequential treatment with IV levetiracetam, valproic acid and phenobarbital.

CSF analysis showed lymphocytic pleocytosis and elevated protein levels, but no pathogens or autoantibodies were identified in the CSF. The initial brain MRI revealed no significant findings. The patient received IV methylprednisolone (1 g daily for 5 days). MRI was repeated at day 19, revealing bilateral hemispheric FLAIR hyperintensities and right frontal leptomeningeal changes, the latter thought to be postictal in nature (Figure 1, panel C). Treatment with IVIG, acyclovir, and broad‐spectrum antibiotics for suspected autoimmune/inflammatory or infectious meningoencephalitis ensued (see Data S1 for details). Due to persistent SE, cenobamate was initiated with titration following the manufacturer's recommended biweekly schedule. Three days later, her EEG showed diffuse slowing with intermittent polymorphic sharp‐wave abnormalities. Propofol was weaned off without recurrence of an ictal pattern. However, her mental status remained poor (GCS 5). Palliative care was consulted, and the family decided to take her home. She left the hospital after 44 days on cenobamate, levetiracetam, phenobarbital, and valproate. The patient passed away 10 days after being discharged.

### 2.4 Case 3

A previously healthy 27‐year‐old female presented with convulsive SE after a case of pharyngitis complicated by fever and abdominal pain. Tonic–clonic seizure activity persisted despite IV diazepam, valproic acid, phenobarbital, phenytoin, brivaracetam and lacosamide. A diagnosis of febrile infection‐related epilepsy syndrome (FIRES) was made. The patient was transferred to the ICU and started on propofol and midazolam. EEG showed generalized ictal discharges with frontotemporal predominance (Figure 2, panel C), prompting the initiation of ketamine and IVIG. Initial brain MRI and CSF studies were unremarkable. Serum *Coxiella burnetii* IgM was positive, prompting a 3‐week course of sulfamethoxazole–trimethoprim, hydroxychloroquine, and doxycycline. In parallel, she received IV methylprednisolone. Weaning of anesthetics resulted in clinical seizure recurrence, so phenytoin and perampanel were added. Repeat brain MRI at day 10 showed interval development of mesial temporal hyperintensities (Figure 1, panel D). On day 13, the patient started ketogenic diet and continued for 9 days with ongoing SE on EEG. Cenobamate was started on day 27 at 12.5 mg daily, and the dose was titrated up every week (instead of the standard 2 weeks recommended by the manufacturer). One week after the initiation of cenobamate, anesthetics were weaned off without clinical or electrographic seizure recurrence (Figure 2, panel D). She remained hospitalized for two additional months and left for rehabilitation on cenobamate, lacosamide, phenobarbital, and valproate.

After discharge, the patient experienced recurrent focal seizures with impaired consciousness, prompting an increase in cenobamate to 200 mg/day. Over the subsequent year, she experienced approximately four to six brief focal seizures with preserved consciousness per month but reported a significant improvement in quality of life, including returning to work, with no major motor or cognitive deficits.

## 3 DISCUSSION

We presented three cases of new‐onset super‐refractory status epilepticus treated with cenobamate. Clinical and electrographic seizure cessation was achieved in all cases shortly after cenobamate initiation at low doses (25–50 mg/day). One patient fully recovered and remains fully independent despite monthly focal seizures with preserved consciousness. The other two were seizure‐free upon discharge; however, their level of consciousness remained poor, and both passed away due to hospitalization‐related complications.

Besides our report, there have been only three cases of cenobamate use in SRSE in the available literature.^8^, ^9^ Carlson et al. described two cases of SRSE that closely resemble our experience with resolution of SRSE at relatively small cenobamate doses and after relatively brief time of being on the medication: in their report, a 45‐year‐old man with FIRES who underwent extensive immunotherapy and antibiotic treatment, and who failed several anesthetics and ketogenic diet, started cenobamate on day 13 at 12.5 mg/day, which was subsequently raised to 50 mg/day in a 2‐week period. SRSE resolved after 5 days of cenobamate 50 mg/day. Their second case, a 52‐year‐old with HSV‐1 encephalitis, received cenobamate at 12.5 mg/day on day 48, titrated to 50 mg/day by day 64, and achieved electrographic seizure resolution between days 52 and 61 with minimal disability reported after discharge.^8^ The third case available in the literature, reported by Falcicchio et al.^9^ is a 26‐year‐old female diagnosed with seronegative autoimmune encephalitis who started cenobamate and an interleukin antagonist on day 34 of SRSE after failing immunotherapy and thiopental coma.^9^ EEG normalization and coma resolution were reported, but the exact timing and the dosing of cenobamate were not detailed (other than a 100 mg loading dose). Regardless, the patient had a good prognosis.^9^

We have added three more cases to the evidence supporting the use of cenobamate as an adjunct treatment of SRSE. Despite status resolution, two of our patients died, which overall falls in line with the expected mortality after SRSE if we consider the three cases available in the literature (see above) that had a good outcome.^4^, ^12^ Potential contributors to the persistent alteration in consciousness and subsequent death could be the very prolonged SRSE in case 1 and the patient's older age in case 2.^13^ The contribution of a probable genetic encephalopathy associated with an SCN7A variant (Case 1) to the patient's outcome is uncertain. We acknowledge that we cannot assess if introducing cenobamate earlier had anything to do with the prognosis in our series or if SRSE resolution can be attributed only to cenobamate vs. the combination of cenobamate with other antiseizure medication as well as immunotherapy; however, it was not until cenobamate initiation that SRSE abated in all three patients, and in Case 3, the only one with a good outcome in our series, cenobamate was started much sooner than in the other two cases.

Why may cenobamate help terminate SRSE? Its mechanism of action includes positive allosteric GABA_A_ modulation as well as inhibition of sodium channels, which would, in theory, target key mechanisms of refractory SE such as loss of GABA_A_ activity, excessive glutamatergic excitation (NMDA‐ and AMPA‐mediated) and persistent sodium current.^14^ Specifically, GABA_A_ allosteric modulation, receptors at a binding site distinct from that of benzodiazepines, may help overcome the benzodiazepine resistance that characterizes SE^15^ and may be the reason why SRSE cessation was seen at low cenobamate doses in our series and the other cases reported so far. Given the high mortality associated with SRSE,^12^ cenobamate's ease of administration through a feeding tube and the relatively low risk of its use at low doses, it may play a larger role in the management of SRSE in the future. Nonetheless, further studies are needed to address several open questions, including the potential for QT interval alteration, the pharmacokinetics of cenobamate in critically ill patients receiving the drug via nasogastric or feeding tube, the safety of accelerated titration schedules, and clinically relevant drug–drug interactions, notably with barbiturates and other enzyme‐inducing agents mediated through CYP pathways.^10^

There are now six cases of SRSE resolution after cenobamate initiation, supporting a potential role for cenobamate as an adjunctive therapy in SRSE management.

## AUTHOR CONTRIBUTIONS

MR and LAZ drafted the manuscript. RM, MR, RI, MP, and CV collected the data. MR, AF, FT, and LAZ prepared the figures and tables. LFI, RM, MK, PC, YM, LAZ, CV, and RI critically revised the manuscript for important intellectual content. LFI, RM, PC, RI, LAZ, MP, YM supervised the study. All authors reviewed the manuscript and approved the final version for submission.

## FUNDING INFORMATION

The authors have nothing to report.

## CONFLICT OF INTEREST STATEMENT

CV has received research support and personal fees from Angelini Pharma, Lundbeck, Jazz Pharmaceuticals, Eisai, and UCB; no other conflicts of interest related to this work are present. MR has received research support and personal fees from Angelini Pharma, Lundbeck, Jazz Pharmaceuticals, Eisai, and UCB; no other conflicts of interest related to this work are present. PC has received research support and personal fees from Angelini Pharma, Lundbeck, Jazz Pharmaceuticals, Eisai, and UCB; no other conflicts of interest related to this work are present. The other authors do not have conflicts of interest related to this manuscript.

## CONSENT STATEMENT

Family members of the patients and patients, when applicable, gave a written informed consent.

**Test yourself**

1. Which or the following mechanisms of action could cenobamate's putative efficacy in the management of super‐refractory status epilepticus (SRSE)?
  - Dopamine reuptake inhibition
  - Positive allosteric GABAA modulation
  - Potassium channel inhibition
  - Calcium channel blockade
2. When is status epilepticus (SE) classified as super‐refractory status epilepticus (SRSE)?
  - When seizures persist for more than 5 min despite two benzodiazepine doses
  - When seizures continue for more than 30 min despite benzodiazepine use and one non‐benzodiazepine antiseizure medication use
  - When SE fails to respond to three or more antiseizure medications within 48 h When EEG shows continuous spike‐wave activity lasting more than 12 h
  - When ictal activity persists or recurs for more than 24 h after initiation of anesthetic therapy
3. True or false: Enteral administration of cenobamate through nasogastric tube or PEG tube has similar bioavailability to intact tablet oral ingestion?
  - True
  - False

*Answers may be found in the* *supporting information*.

## Supporting information


Data S1.

Appendix S1.

## Data Availability

The data that support the findings of this study are available from the corresponding author upon reasonable request.
