# Supplementary material for: Cenobamate use in super‐refractory status epilepticus: A report of three cases
Source: Epileptic Disord. 2026 Jul 4;28(5):1661–8. doi: 10.1002/epd2.70270 (PMC13695472; doi:10.1002/epd2.70270)
Supplement: Supplementary file 1 [file EPD2-28-1661-s001.docx]

**Supplementary material**

1. **Testing specifics**
   1.1. **Pathogens in CSF tests –** Meningitis/encephalitis Biofire^®^ Filmarray^®^ panel (bioMérieux, SA, Marcy-l'Étoile, France), which uses multiplex PCR technology for pathogen identification:

- *Escherichia coli K1*
- *Haemophilus influenzae*
- *Listeria monocytogenes*
- *Neisseria meningitidis*
- *Streptococcus agalactiae*
- *Streptococcus pneumoniae*
- Cytomegalovirus (CMV)
- Enterovirus (EV)
- Herpes simplex virus 1 (HSV-1)
- Herpes simplex virus 2 (HSV-2)
- Human herpesvirus 6 (HHV-6)
- Human parechovirus (HPeV)
- Varicella zoster virus (VZV)
- *Cryptococcus neoformans/gattii*
  1.2. **Autoantibodies tested on CSF -** Autoimmune Encephalitis Mosaic 6 EURO Pattern, (EUROIMMUN, Lübeck, Germany), which uses transfected cell-based assay to determine:
- Glutamate receptor (type NMDA)
- Contactin-associated protein 2 (CASPR2)
- Glutamate receptors (type AMPA1/2)
- Leucine-rich glioma-inactivated protein 1 (LGI1)
- Immunoglobulin-like family containing LSAMP, OBCAM, and NTM 5 (IgLON5)
- Dipeptidyl aminopeptidase-like protein 6 (DPPX)
- GABA B receptor
- Indirect Immunofluorescence assay on frozen section of mouse brain (in-house tissue-based assay) was performed for onconeural antibody detection.

2. **Treatment specifics**
   2.1. **Immunotherapy schedules**

- Intravenous methylprednisolone: 1g/d for 5 days
- Intravenous immunoglobulin: 2 g/kg intravenously in continuous infusion with rate determined by patient’s tolerance (blood pressure, rashes, etc.)
- Plasma exchange: one session every other day for a total of 5 sessions with a solution of 4-5% albumin as replacement human serum albumin in saline as replacement
- Tocilizumab: 8 mg/kg intravenously administered for ≥ 2 cycles at 1-month intervals
  2.2. **Antimicrobial schedules**
- *CNS empiric coverage*
  - Ceftriaxone 2000 mg IV every 12 h,
  - Linezolid 600 mg IV every 12 h OR vancomycin 1000 mg IV every 12 h
  - *Acyclovir* 10 mg/kg IV every 8 hours for 5 days
- Coxiella burnetii treatment for patient 3
  - Sulfamethoxazole-trimethoprim: 100 mg/kg/day + 20 mg/kg/day intravenous every 6 hours
  - Doxycycline: 200 mg/day intravenous
  2.3. **Ketogenic diet**: caloric intake between 25 and 30 kilocalories per kilogram of body weight (kcal/kg) at a 4:1 or 3:1 ratio guided by a dietitian.
