# Supplementary material for: Cenobamate use in super‐refractory status epilepticus: A report of three cases
Source: Epileptic Disord. 2026 Jul 4;28(5):1661–8. doi: 10.1002/epd2.70270 (PMC13695472; doi:10.1002/epd2.70270)
Supplement: Supplementary file 2 [file EPD2-28-1661-s002.docx]

**TEST YOURSELF**

**Answers**

**1. B**

Correct answer: B. Cenobamate works through positive allosteric GABAA modulation and inhibition of sodium channels and loss of GABAergic activity, excess glutamatergic excitation, and persistent sodium current are characteristic of refractory SE thus making cenobamate an attractive option.

**2. D**

Correct answer: D. SRSE is defined as ictal activity that persists or recurs for more than 24 h after anesthetic therapy has been initiated. It carries a high mortality rate of 22%–32%, and oral add-on agents such as perampanel and topiramate have shown mixed results in its management.

**3. A**

Correct answer: A. True. In the case series, cenobamate was administered via nasogastric tube or percutaneous endoscopic gastrostomy (PEG). Both routes have similar bioavailability to intact tablet ingestion, making them practical and safe options for critically ill patients who cannot swallow.
